# Remote Ischemic Preconditioning in a Cirrhotic Patient Undergoing Major Hepatectomy

**DOI:** 10.7759/cureus.9056

**Published:** 2020-07-07

**Authors:** Emrullah Birgin, Christoph Reissfelder, Nuh Rahbari

**Affiliations:** 1 Department of Surgery, Universitätsmedizin Mannheim, Medical Faculty Mannheim, Heidelberg University, Mannheim, DEU

**Keywords:** cirrhosis, liver failure, hepatoprotection, hepatectomy

## Abstract

Remote ischemic preconditioning (RIPC) has been shown to reduce ischemic reperfusion injury for patients undergoing hepatectomy for colorectal liver metastasis. We present a case of a 69-year-old male who underwent right hepatectomy for a multifocal hepatocellular carcinoma of the right liver and concomitant liver cirrhosis (Child-Pugh stage A). We performed portal vein embolization prior to surgery and intraoperative RIPC of the iliac vessels. The postoperative course after major hepatectomy went uneventful.

## Introduction

Remote ischemic preconditioning (RIPC) is characterized by short episodes of ischemia and reperfusion induced by temporary arterial occlusion of a limb to release cytokines, which mediate protection from ischemic injury in a distant organ of interest (e.g. heart, bowel, or liver) [1,2]. In a randomized trial, RIPC was demonstrated to provide hepatoprotection in patients undergoing hepatectomy for colorectal liver metastasis [3]. However, the hepatoprotective effects of RIPC remain unknown for patients undergoing major hepatectomy in the context of significant cirrhosis. Herein, we report a case of hepatocellular carcinoma (HCC) with underlying liver cirrhosis and intraoperative RIPC of the iliac vessels.

## Case presentation

**Figure 1:**
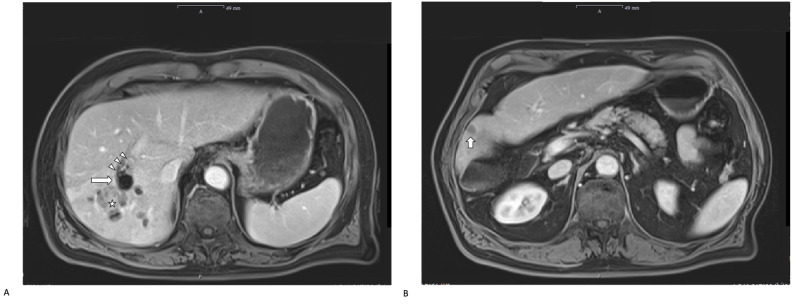
Preoperative imaging Preoperative MRI was performed a week prior to hepatectomy and shows a considerable degree of hypertrophy of the left liver lobe following portal vein embolization. The MRI demonstrates the hepatic lesions in segment V (indicated by the white star, A), and in segment VII (indicated by the white short arrow, B). The white long arrow in 1A indicates microspheres in the right portal vein with adherent thrombotic material (indicated by three white triangles).

A 69-year-old man with alcohol-related liver cirrhosis (Child-Pugh stage A) was referred to our hospital due to an incidental finding of two hepatic masses. Alpha fetoprotein and serology for hepatitis B and C were negative. On MRI, a 3.5 x 3 cm mass was seen in liver segment V at the bifurcation of the right portal pedicle and another 1.5 x 1 cm mass in liver segment VII (Figure 1).

A core needle biopsy of the lesion in segment V confirmed the diagnosis of a moderately differentiated (G2) HCC (Ki67: 20%). At the multidisciplinary board, a right hepatectomy was recommended. Liver volumetry revealed a total liver volume of 1,511 ml and a volume of the future liver remnant of 386 ml (future liver remnant ratio: 25%). The patient’s baseline liver biochemical tests showed normal findings (Table 1).

**Table 1:** Preoperative laboratory tests

Parameters	Result	Reference range
Albumin (g/l)	39.1	34-50
Bilirubin (mg/dl)	0.86	0.2-1.0
Creatinine (mg/dl)	0.92	0.81-1.44
Alkaline phosphatase (U/I)	64	40-130
Gamma-glutamyl transferase (U/I)	62	0-60
Aspartate aminotransferase (U/I])	44	0-50
Alanine aminotransferase (U/I)	32	0-50
White blood count (x10^9^/I)	5.17	4.2-10.2
Hemoglobin (g/dI)	15.0	13.2-16.7
Platelets (x10^9^/I)	147	145-348
International normalized ratio (INR)	1.11	0.9-1.15
Quick (%)	80	70-130
C-reactive protein (mg/l)	<2.9	0-5

**Figure 2:**
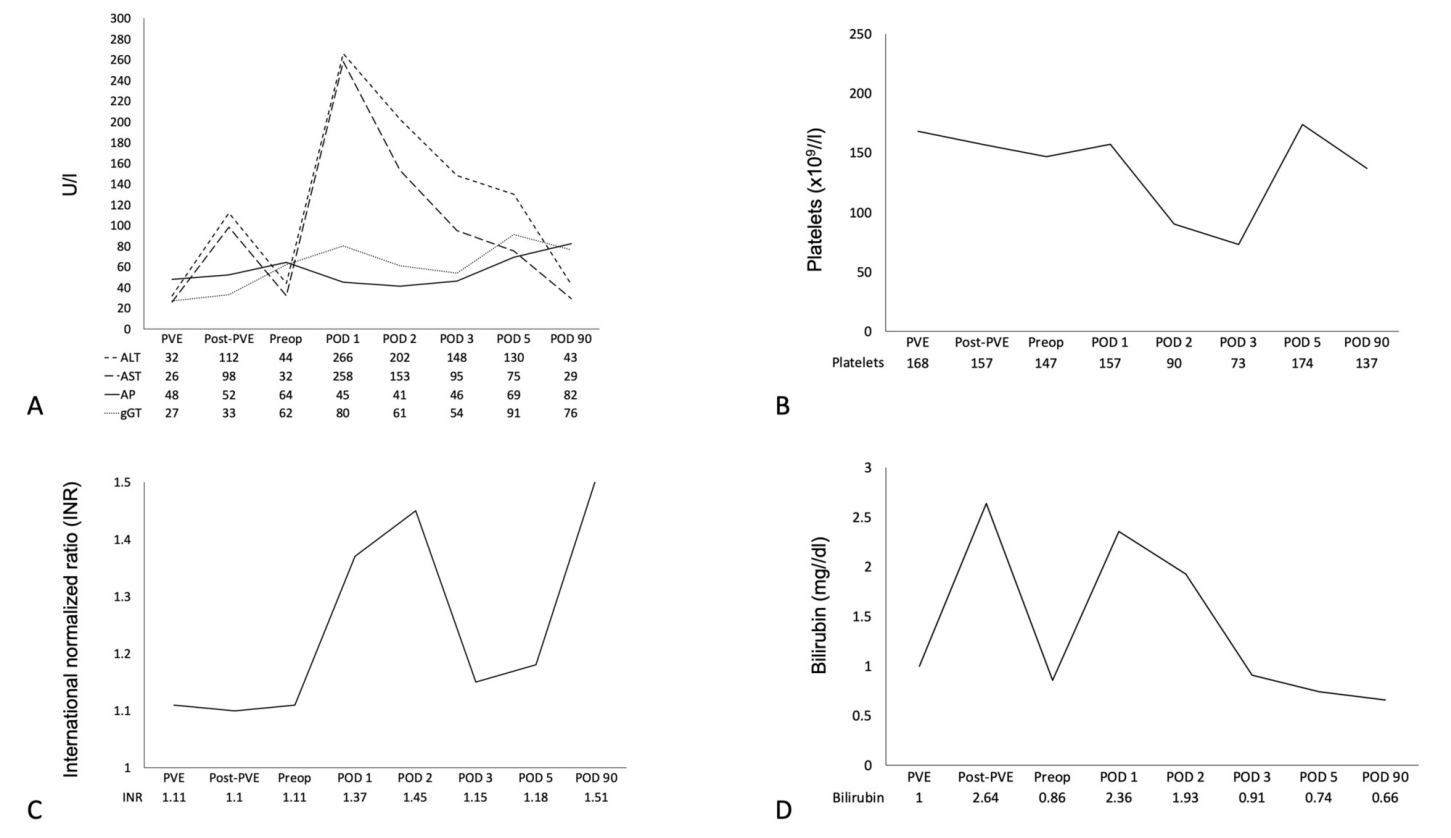
Blood parameters over time ALT, alanine aminotransferase; AST, aspartate aminotransferase; AP, alkaline phosphatase; gGT, gamma-glutamyl transferase; bilirubin; INR, international normalized ratio; platelets. PVE, portal vein embolization; post-PVE, day after portal vein embolization; preop, day before surgery; POD, postoperative day.

Due to the small future liver remnant, the presence of cirrhosis, and the need for a major hepatectomy, preoperative right portal vein embolization (PVE) was performed using 500-700 µm embosphere microspheres. The intervention was uneventful. On the day after the intervention, his total serum bilirubin increased to 2 mg/dl and his transaminases around 100 U/l (Figure 2). 

A follow-up MRI at four weeks post-PVE demonstrated significant hypertrophy of the future liver remnant with a volume of 638 ml and a total liver volume of 1,706 ml (future liver remnant ratio: 37%). However, there was suspicion of microspheres and adhering thrombus protruding into the main portal vein trunk (Figure 1). 

For this reason, intraoperative exploration of the portal vein trunk was planned. As prolonged hepatic ischemia was anticipated in the case of vascular reconstruction of the portal vein, the decision was made to apply RIPC. 

**Figure 3:**
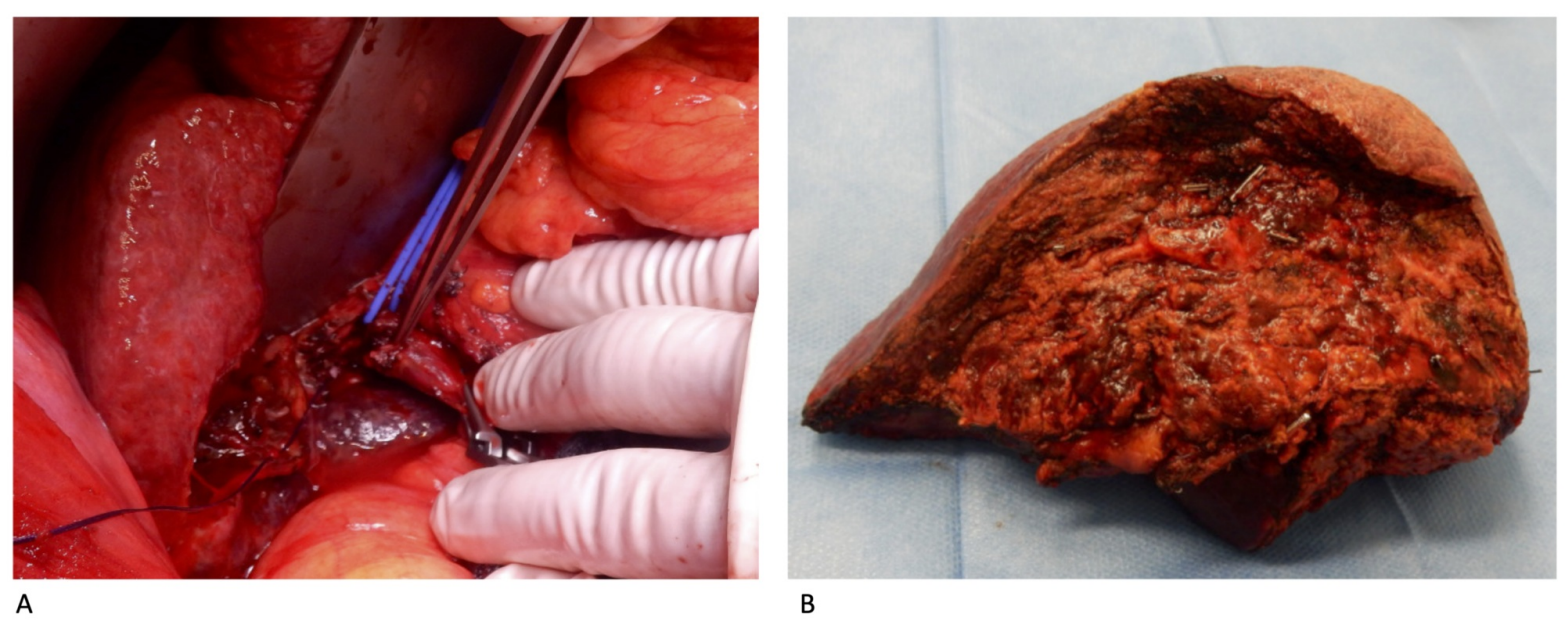
Hepatectomy (A) Around the left hepatic pedicle a vessel sling was placed. After clamping the portal vein both proximally and distally, the thrombosis was removed through venotomy of the right portal vein. The thrombotic material is demonstrated at the tip of the forceps. (B) Resected specimen.

The patient was taken to the operating room five weeks after right PVE. After induction of anesthesia and before laparotomy, three cycles of ischemia and reperfusion (three 10-minute inflations and deflations) were induced via a tourniquet on the patients’ right thigh that was intermittently inflated to a pressure of 100 mmHg above the baseline systolic blood pressure of the patient. Remarkably, the patient responded to the episodes of reperfusion with a transient drop of his blood pressure (mean relative drop of systolic blood pressure of 13% and mean relative drop of diastolic blood pressure of 8%), indicating the effectiveness of the intervention [3]. No complications were noted due to the application of RIPC. All three portal vein vessels were clamped with vascular clamps and a venotomy of the main portal vein trunk was carried out (Figure 3).

The thrombus was found to be adherent to the main portal vein wall. After it was peeled off the portal vein wall, the main portal vein was closed resulting in takedown of the right portal vein. The cumulative duration of portal vein occlusion was 25 minutes. Subsequently, the right hepatic artery was suture ligated. The liver parenchymal transection was performed using an energy device and intermittent portal triad clamping (PTC) for a cumulative duration of 30 minutes. The right hepatic pedicle was mass transected and the resection completed with takedown of the right hepatic vein using a stapling device. No drains were placed and the abdomen was closed in a regular fashion. The total operating time was 281 minutes, and the total amount of intraoperative blood loss was 500 ml. 

After the surgery, the patient was monitored on an intermediate care unit for 24 hours before he was transferred to the regular ward. The entire postoperative course was uneventful. Remarkably, serial analyses of his biochemical liver tests demonstrated no impairment of his postoperative liver function. Of particular interest, there was only a mild increase in his transaminase levels, bilirubin level, platelets, and international normalized ratio (INR) as markers of hepatic parenchymal injury (Figure 2). Peak values of serum alanine aminotransferase (ALT) and aspartate aminotransferase (AST) were already observed 24 hours postoperatively; however, these values almost normalized over time (Figure 2). The patient was discharged on postoperative day 8. At one-, three-, and six-month follow-up, the patient had a normal liver function and there were no signs of recurrence.

## Discussion

Surgical resection remains the primary curative therapy for patients with HCC [4]. However, up to 40% of patients with HCC present with concomitant portal vein thrombosis at the time of diagnosis and are rarely considered as candidates for surgical resection or even for further treatment [5]. Furthermore, a significant percentage of patients with HCC suffer from liver cirrhosis of varying severity. Cirrhotic patients undergoing hepatic resection are at high risk of postoperative morbidity and in particular posthepatectomy liver failure (PHLF) [6]. In fact, major hepatectomy is not feasible in many cirrhotic patients with HCC due to their liver’s low functional reserve. Intraoperative hemorrhage is known to further increase the risk of PHLF in cirrhotic patients undergoing major hepatectomy [7,8]. PTC might be an effective measure to reduce the amount of intraoperative blood loss. However, there is substantial evidence that the cirrhotic liver is more susceptible to ischemia reperfusion injury (IRI) that might put patients at risk for inadequate postoperative liver regeneration and PHLF [9]. This is of particular importance in patients requiring complex resections with or without vascular reconstruction. Commonly applied methods to minimize IRI include intermittent PTC and ischemic preconditioning with a brief period of hepatic inflow occlusion and reperfusion followed by a prolonged period of PTC [10]. Recently, the concept of RIPC has gained increasing attention in the literature. RIPC was initially introduced in 1993 in a rodent cardiac surgery model and since numerous preclinical studies proved a protective effect for RIPC against IRI in various organ systems of mammals, including the liver, heart, brain, and intestine [11-13]. Although the benefit of RIPC to reduce myocardial injury after cardiac surgery was also shown in various clinical trials, two large randomized controlled trials failed to show an improved clinical outcome [14-17]. A recent randomized controlled feasibility study demonstrated RIPC as a valuable method to reduce liver injury in patients undergoing hepatectomy for colorectal liver metastasis without steatosis or fibrosis [3]. Kanoria et al. induced RIPC by a pneumatic tourniquet around the upper thigh for 60 minutes (three 10-minute inflations and deflations). Twenty-four hours after hepatectomy, the mean serum transaminase levels were almost 50% lower in patients with the preconditional stimulus compared to patients without preconditioning [3]. Nevertheless, another recent trial failed to show a statistically significant decrease of serum transaminase levels 24 hours after hepatectomy in patients with HCC and RIPC compared to sham treatment [18]. But none of these patients underwent PVE or had concomitant portal vein thrombosis before surgery. Furthermore, the trial used a different RIPC protocol with a pneumatic tourniquet around the upper arm for 20 minutes (four 5-minute inflations and deflations) and the majority of the included patients underwent only minor hepatectomies. Thus, one has to consider those different methods of RIPC were applied in all these trials (e.g. duration of vascular occlusion, the total number of cycles, direct vascular clamping vs limb tourniquet) prohibiting a direct comparison and conclusion of these studies.

In our case report, we observed similar transaminase levels 24 hours after major hepatectomy in a patient with Child-A liver cirrhosis demonstrating RIPC to be feasible and effective in a pre-damaged liver with portal vein thrombosis. Although the underlying mechanisms of RIPC are not fully understood, RIPC was identified to induce systematic serotonin release of platelets which induces endothelial stimulation of vascular endothelial growth factor (VEGF) [19]. Nevertheless, the molecular mechanisms of RIPC in underlying fibrosis or cirrhosis are unclear. Of note, one has to consider that primary liver tumors are also able to produce VEGF, potentially, interfering with VEGF-dependent effects by RIPC [20].

## Conclusions

To the best of our knowledge, this is the first report of RIPC in a patient with HCC and concomitant portal vein thrombosis. The uneventful postoperative course after major hepatectomy suggested that RIPC was beneficial even after PVE and intraoperative portal vein reconstruction. RIPC reflects a promising method in patients with impaired liver function. As multimodal therapeutic approaches in HCC are emerging, RIPC seems to be another new modality to provide safe hepatic resections. However, further prospective trials are required to evaluate the definitive role of RIPC in patients undergoing major hepatectomy.
